# TRPV1 is crucial for proinflammatory STAT3 signaling and thermoregulation-associated pathways in the brain during inflammation

**DOI:** 10.1038/srep26088

**Published:** 2016-05-18

**Authors:** Ayaka Yoshida, Eriko Furube, Tetsuya Mannari, Yasunori Takayama, Hiroki Kittaka, Makoto Tominaga, Seiji Miyata

**Affiliations:** 1 Department of Applied Biology, Kyoto Institute of Technology, Matsugasaki, Sakyo-ku, Kyoto 606-8585, Japan; 2 Division of Cell Signaling, Okazaki Institute for Integrative Bioscience, (National Institute for Physiological Sciences), National Institute of Natural Sciences, Okazaki, Aichi 444-8787, Japan

## Abstract

Transient receptor potential vanilloid receptor 1 (TRPV1) is a non-selective cation channel that is stimulated by heat (>43 °C), mechanical/osmotic stimuli, and low pH. The importance of TRPV1 in inflammatory responses has been demonstrated, whereas its participation in brains remains unclear. In the present study, the intracerebroventricular (icv) administration of the TRPV1 agonist resiniferatoxin (RTX) induced the activation of signal transducer and activator of transcription 3 (STAT3) in circumventricular organs (CVOs) and thermoregulation-associated brain regions with a similar patttern to the peripheral and icv administration of lipopolysaccharide (LPS). With the peripheral and icv LPS stimuli, STAT3 activation was significantly lower in *Trpv1*^−/−^ mice than in *Trpv1*^+/+^ mice. The icv administration of RTX induced transient hypothermia, whereas that of the TRPV1 antagonist capsazepine enhanced the magnitude and period of LPS-induced hyperthermia. These results indicate that TRPV1 is important for activating proinflammatory STAT3 signaling and thermoregulation-associated brain pathways in the brain.

Transient receptor potential vanilloid 1 (TRPV1) is a non-selective cation channel with a preference for Ca^2+^ and is activated by heat, mechanical/osmotic stimuli, protons, and pH < 5.9^1^,^2^. TRPV1 is strongly expressed in the terminals of the dorsal root ganglion and trigeminal ganglion and in some peptidergic sensory neurons in the nodose ganglion^1^,^2^. These TRPV1-expressing sensory neurons innervate the skin and visceral organs and have nociceptive or pain functions through the activation of the primary afferent fibers innervating them^3^,^4^.

TRPV1 is activated by the plant-derived lipophilic substances capsaicin and resiniferatoxin (RTX), which show structural similarities to the endogenous TRPV1 agonists of fatty acid derivatives^5^,^6^,^7^. The peripheral administration of the TRPV1 agonists capsaicin, RTX, rinvanil, and arvanil has been shown to induce severe hypothermia associated with skin vasodilatation^8^,^9^,^10^,^11^,^12^; however, this did not occur in *Trpv1*^−/−^ mice^13^. Hot peppers have been used in diets and medicinal treatments worldwide in order to reduce body temperature^14^. Capsaicin crosses the blood-brain barrier (BBB)^15^ and the dose needed to induce hypothermia is at least 25-fold higher for peripheral administration (~5 μg)^16^ than for central administration (200 ng)^17^. Moreover, the intracerebroventricular (icv) administration of the TRPV1 agonists capsaicin^8^,^9^, RTX^18^, and arvanil^19^ has been shown to induce transient hypothermia. Therefore, capsaicin is assumed to act on thermoregulation-associated brain regions to cause hypothermia^20^,^21^; however, the mechanisms responsible for the effects of TRPV1 agonists in the brain remain unknown.

The peripheral administration of various TRPV1 antagonists causes hyperthermia with tail skin vasoconstriction and/or the activation of thermogenesis^10^,^13^. However, previous studies reported that the icv administration of TRPV1 antagonists failed to induce hyperthermia under normal conditions^13^,^22^. *Trpv1*^−/−^ mice showed similar increases in body temperature against standard heat loading, but exhibited a higher amplitude for the daily body temperature rhythm^23^. TRPV1 antagonists may cause hyperthermia by disrupting the peripheral suppression mechanisms of hyperthermia^5^,^10^,^23^. This effect precludes the development of TRPV1 antagonists, widely regarded as next-generation pain therapeutics^7^,^10^.

Sensory circumventricular organs (CVOs) include the organum vasculosum of the lamina terminalis (OVLT), subfornical organ (SFO), and area postrema (AP). They have high vascular permeability due to the lack of the BBB at endothelial tight junctions, are considered to act as the main entry route of blood-derived inflammatory cytokines and pathogens^24^,^25^,^26^, and convey their information into inflammatory and thermoregulatory brains regions^27^,^28^. In sensory CVOs, glial fibrillary acidic protein (GFAP)-expressing multipotent neural stem cells (NSCs) express TRPV1 and NaX that sense and control body fluid homeostasis^25^,^29^. The expression levels of Toll-like receptor 4 (TLR4) and its co-receptor CD14 were found to be markedly higher in sensory CVOs than in other brain regions^30^,^31^,^32^. The peripheral and central administration of lipopolysaccharide (LPS) was recently shown to stimulate TLR4 on the multipotent NSCs of sensory CVOs, which resulted in faster proinflammatory responses such as the activation of JAK-signal transducer and activator of transcription 3 (STAT3) and nuclear factor-κB (NF-κB) in CVOs than in other brain regions^27^,^33^,^34^. These findings strongly suggest that blood-derived LPS and cytokines initially reach CVOs, which lack the BBB, and induce the early phase of inflammatory responses; information is then transferred to adjacent thermoregulatory brain regions^27^,^35^.

However, progress in understanding the potential involvement of TRPV1 in inflammatory and thermoregulatory responses in the brain has been limited. Therefore, we herein used genetically modified mice and pharmacological tools to determine the contribution of TRPV1 in the brain to inflammatory and thermoregulatory actions. Our results demonstrated that LPS-induced STAT3 activation was deficient in *Trpv1*^−/−^ mice and also that the TRPV1 agonist RTX activates STAT3 in sensory CVOs and thermoregulatory hypothalamic regions. The TRPV1 agonist RTX tonically induced hypothermia in a dose-dependent manner under normal conditions, while the TRPV1 antagonist capsazepine augmented the magnitude and period of LPS-induced hyperthermia. These results indicate that TRPV1 in the brain is inactive under normal conditions, but is activated under inflammatory conditions, and the TRPV1 signaling cascade activates proinflammatory STAT3 signaling and endogenous antipyretic thermoregulatory pathways.

## Results

### Deficiency in brain STAT3 activation in *Trpv1* ^−/−^ mice during inflammation

**Figure 1:**
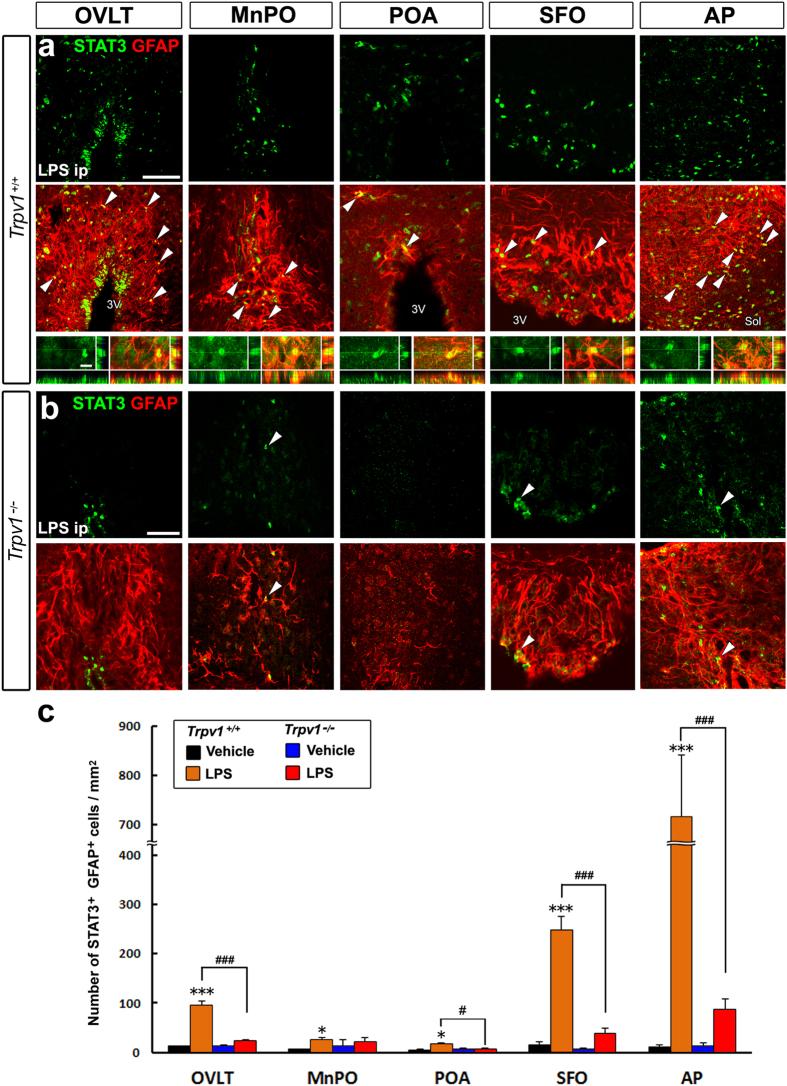
A deficiency in STAT3 activation in GFAP^+^ NSCs and astrocytes in *Trpv1*^−/−^ mouse brains upon the peripheral LPS stimulation. C57BL/6J mice received an intraperitoneal administration of 50 μg/kg LPS and were sacrificed for STAT3 immunohistochemistry. (**a,b**) The nuclear translocation of STAT3 was often observed in GFAP^+^ NSCs in sensory CVOs (OVLT, SFO, and AP) and thermoregulatory hypothalamic subregions (MnPO and POA) in *Trpv1*^+/+^ mice 2 hr after the peripheral LPS stimulation (top and middle panels in **a**), while it was rarely observed in Trpv1^−/−^ mice (**b**). A three-dimensional image analysis demonstrated the presence of STAT3^+^ nuclei in GFAP^+^ NSCs and astrocytes (bottom panels in **a**). Arrowheads indicate STAT3^+^ nuclei in GFAP^+^ cells. Scale bars = 50 (top panels of **a**,**b**) and 10 (bottom panels of **a**) μm. (**c**) The quantitative analysis showed that the number of STAT3^+^ GFAP^+^ cells in Trpv1^+/+^ mice was significantly increased in sensory CVOs and hypothalamic thermoregulatory brain subregions after the intraperitoneal administration of 50 μg/kg LPS, but was not in Trpv1^−/−^ mice. Sol, solitary nucleus; 3V, 3^rd^ ventricle. Data (n = 4) were expressed as the mean (±s.e.m.). ^*^P < 0.05, ^***^P < 0.001 vs the vehicle in Trpv1^+/+^ mice and ^#^P < 0.05, ^###^P < 0.001 between Trpv1^+/+^ and Trpv1^−/−^ mice by ANOVA with Turkey’s *post hoc* tests.

We determined whether TRPV1 plays a role in inflammatory signaling cascades in the brain. STAT3 is a well-known marker of proinflammatory responses in the brain^27^,^34^. The activation of STAT3 may be detected by the phosphorylation of STAT3 and its subsequent nuclear translocation^36^. We previously demonstrated that GFAP^+^ multipotent NSCs in sensory CVOs expressed TRPV1 and TLR4^25^,^29^,^32^ and also revealed the activation of STAT3 following an LPS-induced inflammatory stimulation. STAT3 activation occurred in GFAP^+^ NSCs in sensory CVOs and GFAP^+^ astrocytes in thermoregulatory hypothalamic subregions such as the median preoptic nucleus (MnPO) and preoptic area (POA) in *Trpv1*^+/+^ mice after the peripheral administration of 50 μg/kg LPS (Fig. 1a). The intraperitoneal administration of LPS has been shown to induce hyperthermia at doses of 10~100 μg/kg in mice^37^. STAT3 activation is reportedly absent in unstimulated control mice (data not shown)^32^. STAT3 signaling activation has also been detected in the ventral hippocampal commissure (vhc), median eminence (ME), arcuate nucleus (Arc), supraoptic nucleus (SON), and solitary nucleus (Sol) in *Trpv1*^+/+^ mice (Fig. 1a and Fig. S1a–d). In contrast to *Trpv1*^+/+^ mice, prominent STAT3 activation was hardly detected in any brain region including sensory CVOs and thermoregulatory brain subregions in *Trpv1*^−/−^ mice after the peripheral administration of LPS (Fig. 1b and Fig. S1e–h). The peripheral administration of 50 μg/kg LPS clearly increased the number of STAT3^+^ GFAP^+^ NSCs in sensory CVOs (P < 0.001) and STAT3^+^ GFAP^+^ astrocytes in the MnPO and POA (P < 0.05) in *Trpv1*^+/+^ mice more than that of the control (Fig. 1c). However, the number of STAT3^+^ GFAP^+^ NSCs and astrocytes in these brain regions in *Trpv1*^−/−^ mice was not significantly affected by the peripheral administration of LPS (P > 0.05).

**Figure 2:**
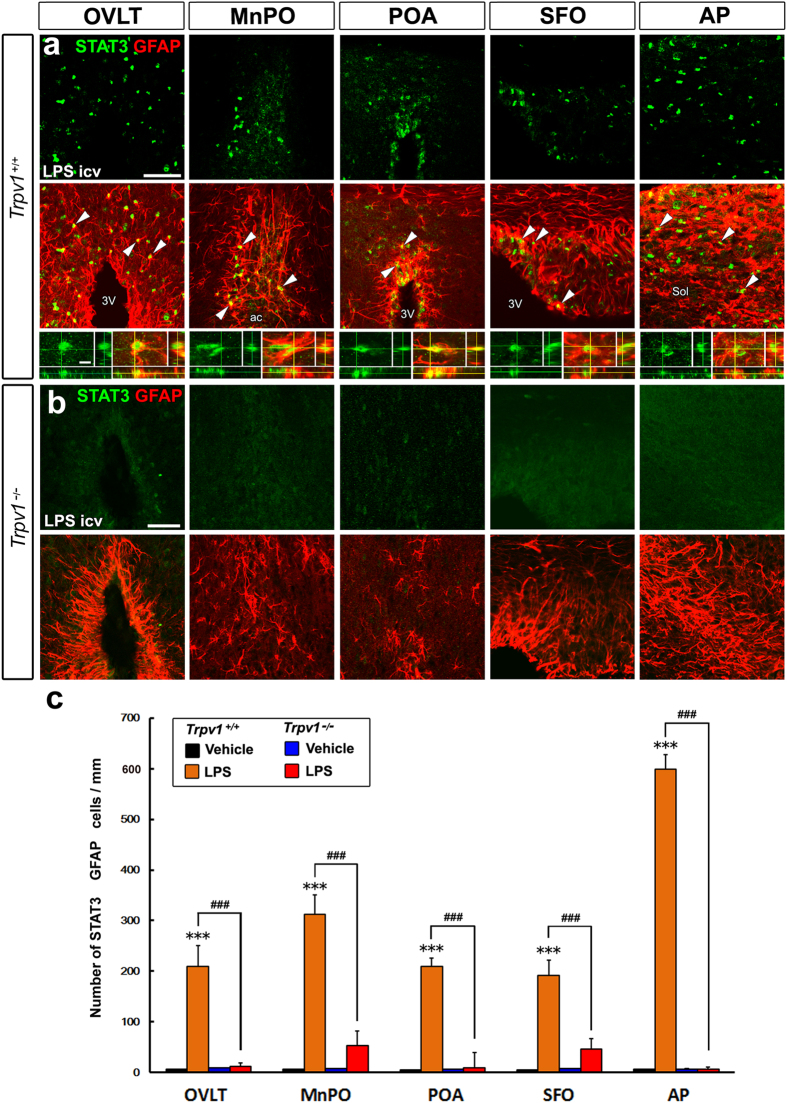
The lack of STAT3 activation in GFAP^+^ NSCs and astrocytes in Trpv1^−/−^ mouse brains during inflammation. C57BL/6J mice received an icv administration of 30 ng/kg LPS and were sacrificed for STAT3 immunohistochemistry. (**a**,**b**) The nuclear translocation of STAT3 was observed in a large number of GFAP^+^ NSCs in sensory CVOs and in GFAP^+^ astrocytes in thermoregulatory hypothalamic subregions in Trpv1^+/+^ mice 2 hr after the central LPS stimulation (top and middle panels in **a**), while the translocation of STAT3 was scarcely detected in Trpv1^−/−^ mouse brains (**b**). A three-dimensional image analysis demonstrated the occurrence of STAT3^+^ nuclei in GFAP^+^ NSCs and astrocytes (bottom panels in **a**). Arrowheads indicate STAT3^+^ nuclei in GFAP^+^ cells. Scale bars = 50 (top panels of **a**,**b**) and 10 (bottom panels of **a**) μm. (**c**) The quantitative analysis revealed that the number of STAT3^+^ GFAP^+^ cells in Trpv1^+/+^ mouse brains was markedly elevated in sensory CVOs and hypothalamic thermoregulatory brain subregions after the central administration of 30 ng/kg LPS, while this number did not increase in Trpv1^−/−^ mice. ac, anterior commissure; 3V, 3^rd^ ventricle; Sol, solitary nucleus. Data (n = 4) were expressed as the mean (±s.e.m.). ^***^P < 0.001 vs the vehicle in Trpv1^+/+^ mice and ^###^P < 0.001 between Trpv1^+/+^ and Trpv1^−/−^ mice by ANOVA with Turkey’s *post hoc* tests.

In order to clarify whether the deficiency in LPS-induced STAT3 activation in the *Trpv1*^−/−^ mouse is due to a dysfunction in TRPV1 in the brain rather than in the periphery, we examined the effects of the icv administration of LPS on STAT3 activation in the brain. The icv administration of 30 ng/kg LPS strongly induced the activation of STAT3 in GFAP^+^ NSCs in sensory CVOs and GFAP^+^ astrcoytes in the hypothalamic and medulla subregions in *Trpv1*^+/+^ mice, similar to the peripheral administration (Fig. 2a and Fig. S2). However, STAT3 activation was scarcely observed in *Trpv1*^−/−^ mice after the icv administration of LPS (Fig. 2b). No significant differences were observed in the number of STAT3^+^ GFAP^+^ NSCs and astrocytes in *Trpv1*^−/−^ mice between the icv administration of LPS and the vehicle control (Fig. 2c). The brain subregions in which STAT3 activation occurred in GFAP^+^ cells were similar following the peripheral and icv administration of LPS (Table 1). Taken together, these results indicate that TRPV1 is necessary for activating STAT3 in the brain under LPS-induced inflammatory conditions.

### Activation of STAT3 by TRPV1

**Figure 3:**
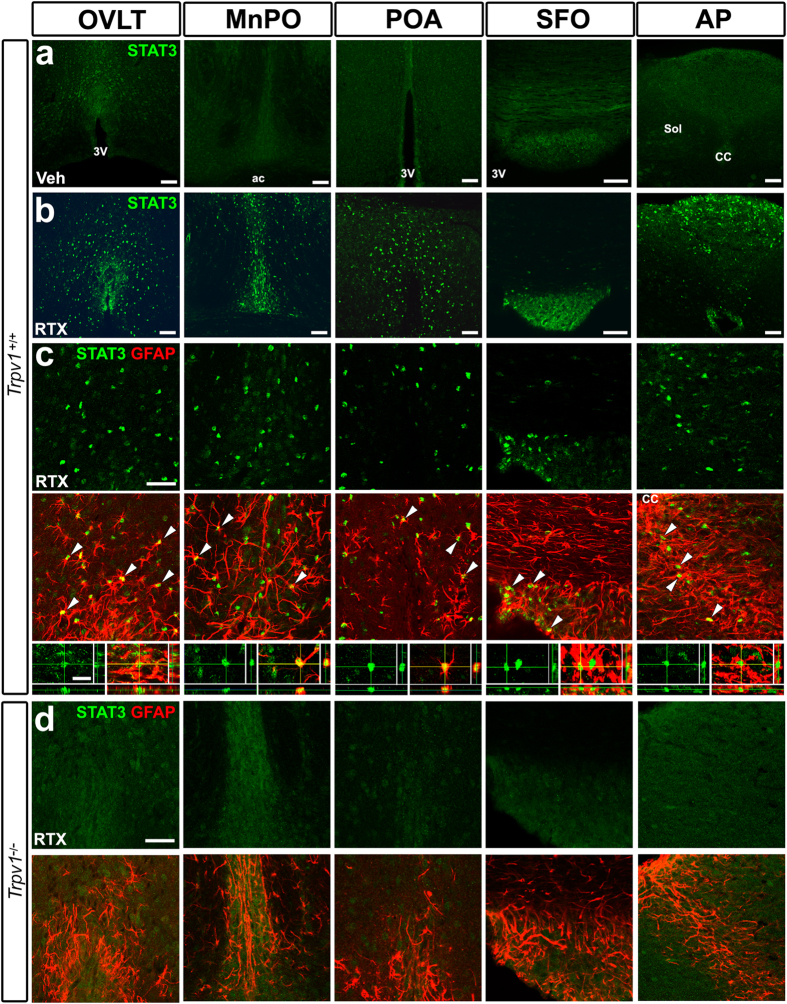
The TRPV1 stimulation leads to STAT3 activation in GFAP^+^ NSCs in sensory CVOs and in GFAP^+^ astrocytes in thermoregulatory hypothalamic subregions. Mice received an icv administration of RTX at a dose of 500 ng/kg and were sacrificed for immunohistochemistry. The central administration of RTX strongly induced the nuclear translocation of STAT3 in sensory CVOs such as the OVLT, SFO, and AP, and thermoregulatory hypothalamic subregions such as the MnPO and POA in Trpv1^+/+^ mice (**a**). Double labeling immunohistochemistry revealed the presence of STAT3^+^ nuclei in GFAP^+^ NSCs in sensory CVOs and GFAP^+^ astrocytes in thermoregulatory hypothalamic brain subregions (**b**). The nuclear translocation of STAT3 was not observed in Trpv1^−/−^ mice (**c**). ac, anterior commissure; CC, central canal; 3V, 3^rd^ ventricle; Sol, solitary nucleus. Scale bars = 50 (**a**, top panels of **b**,**c**) and 10 (bottom panels of **b**) μm.

**Figure 4:**
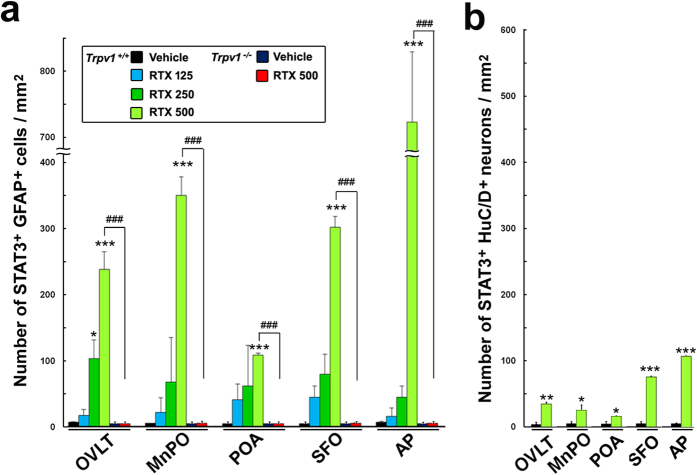
A quantitative analysis showed that the number of STAT3^+^ GFAP^+^ cells in Trpv1^+/+^ mice was significantly increased in sensory CVOs and hypothalamic thermoregulatory subregions after the brain administration of 500 ng/kg RTX. On the other hand, the brain administration of 500 ng/kg RTX did not elevate their number in Trpv1^−/−^ mice. The number of STAT3^+^ HuC/D^+^ neurons was also increased in Trpv1^+/+^ mice, but was markedly lower than that in GFAP^+^ cells. Data were expressed as the mean (±s.e.m.) number of STAT3^+^ cells per 1 mm^2^. *P < 0.05, **P < 0.01, ***P < 0.001 vs the vehicle in Trpv1^+/+^ mice or ^###^P < 0.001 between Trpv1^+/+^ and Trpv1^−/−^ mice by ANOVA with Turkey’s *post hoc* tests or the Student’s *t*-test (n = 4).

TRPV1 activation in the brain by the TRPV1 agonists capsaicin^8^,^9^, RTX^18^, and arvanil^19^ is known to induce transient hypothermia; however, the mechanisms by which the activation of TRPV1 in the brain causes hypothermia, particularly the TRPV1-responsive brain subregions, cellular phenotypes, and signaling pathways, currently remain unclear. The icv administration of the TRPV1 agonist RTX more strongly induced STAT3 activation in sensory CVOs and thermoregulatory hypothalamic subregions at a dose of 500 ng/kg (Fig. 3b) than the vehicle (Fig. 3a). Double labeling immunohistochemistry showed that STAT3 activation occurred in GFAP^+^ NSCs in sensory CVOs and GFAP^+^ astrocytes in thermoregulatory hypothalamic subregions in *Trpv1*^+/+^ mice (Fig. 3c). RTX also activated STAT3 in GFAP^+^ astrocytes in the vhc, ME, Arc, and Sol in *Trpv1*^+/+^ mice (Fig. S3a–d). It is important to note that the brain subregions in which RTX-induced STAT3 activation occurred in GFAP^+^ cells were similar to those following the peripheral and icv administration of LPS (Table 1). STAT3 activation was never detected in sensory CVOs or thermoregulatory hypothalamic subregions in *Trpv1*^−/−^ mice after the icv administration of 500 ng/kg RTX (Fig. 3d). The results of the quantitative analysis showed that the icv administration of 500 ng/kg RTX significantly increased the number of STAT3^+^ GFAP^+^ cells in *Trpv1*^+/+^ mice (P < 0.001), while that of 125 and 250 ng/kg RTX slightly increased this number, except for in the OVLT (P > 0.05, Fig. 4a). The icv administration of 500 ng/kg RTX slightly increased the number of STAT3^+^ HuC/D^+^ neurons (Fig. 4b and Fig. S4).

NF-kB is the main downstream signaling pathway of the LPS receptor TLR4 and, thus, is often used as a marker of proinflammatory responses^38^. The icv administration of 500 ng/kg RTX did not induce the activation or nuclear translocation of NF-kB, whereas the peripheral administration of 50 μg/kg LPS induced NF-kB activation in sensory CVOs and thermoregulatory hypothalamic subregions (Fig. S5). Collectively, these results indicate that the TRPV1 signal cascade leads to the activation of STAT3 in GFAP^+^ cells in sensory CVOs and thermoregulatory hypothalamic regions in the brain.

### TRPV1-dependent control of body temperature

**Figure 5:**
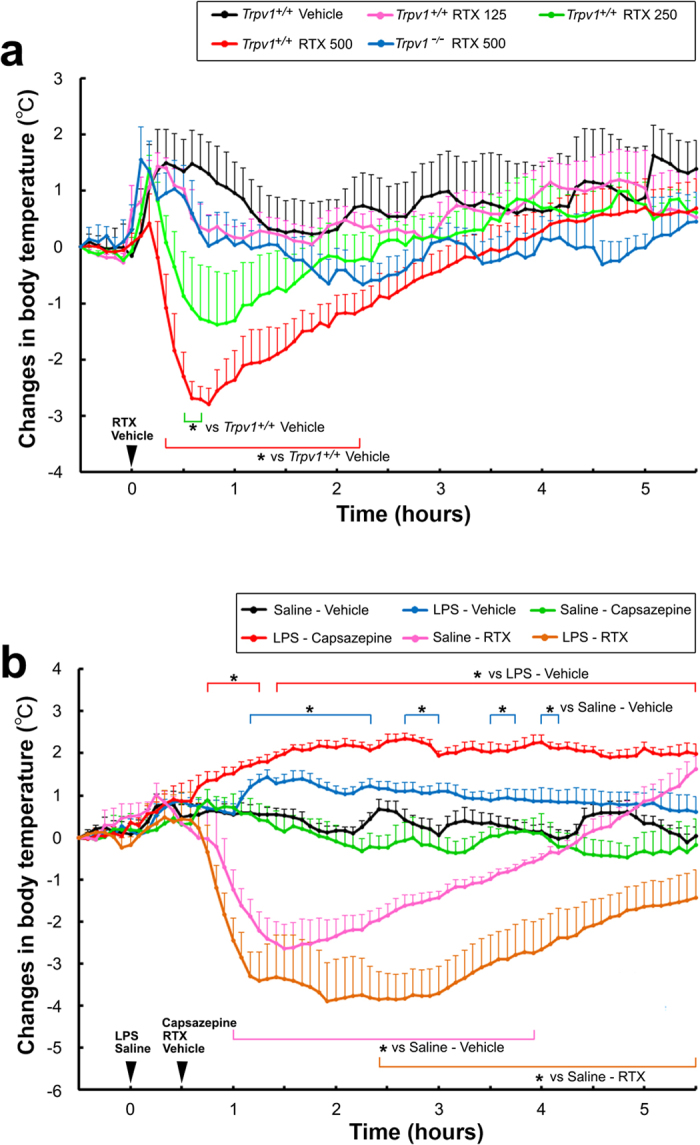
Courses of abdominal temperatures after the administration of the TRPV1 agonist RTX or antagonist capsazepine under normal and LPS-induced inflammatory conditions. Body temperature was measured with a G2 E-mitter transponder implanted intraperitoneally. (**a**) The brain administration of 125 ~ 500 ng/kg RTX transiently decreased abdominal core temperature in a dose-dependent manner under normal conditions. RTX reduced body temperature at doses of 250 and 500 ng/kg in Trpv1^+/+^ mice, but not in Trpv1^−/−^ mice. (**b**) LPS-induced hyperthermia was augmented when 100 μg/kg capsazepine was infused into the brain 30 min after the administration of 100 μg/kg LPS. In contrast, RTX-induced hypothermia was facilitated by the administration of 100 μg/kg LPS, whereas LPS alone caused hyperthermia. *P < 0.05, by ANOVA with the Student’s *t*-test. Please refer to detailed changes in body temperature and statistical analyses (Supplemental Table).

We next investigated whether TRPV1 in the brain participates in controlling body temperature under normal and inflammatory conditions. We monitored core body temperature in response to pharmacological treatments in mice fitted with a G2 E-mitter. All animals initially showed stress fever as a consequence of handling during/after the administration procedure regardless of whether LPS or saline was given. The icv administration of 0.9% saline induced a transient increase in core body temperature of approximately 1.50 ± 0.60 °C at 20 min, which soon decreased to the initial body temperature. The icv administration of 250 and 500 ng/kg, but not 125 ng/kg, to *Trpv1*^+/+^ mice decreased body temperature at 35–45 min (P < 0.05 vs vehicle) with a nadir (−1.38 ± 0.94 °C) at 50 min and 25–140 min (P < 0.05 vs vehicle) with a nadir (−2.79 ± 0.28 °C) at 45 min (Fig. 5a). However, the icv administration of 500 ng/kg RTX to *Trpv1*^−/−^ mice induced stress-associated hyperthermia similar to the saline vehicle control, without a subsequent decrease in body temperature (P > 0.05). These results indicate that the activation of TRPV1 by RTX in the brain induces transient hypothermia in a dose-dependent manner under normal conditions.

We then examined the effects of the TRPV1 antagonist capsazepine and agonist RTX on body temperature under LPS-induced inflammatory conditions (Fig. 5b). The icv administration of the TRPV1 antagonist capsazepine at a dose of 100 μg/kg and agonist RTX at a dose of 500 ng/kg to *Trpv1*^+/+^ mice was performed 30 min after the intraperitoneal administration of 50 μg/kg LPS. The peripheral administration of LPS elevated body temperatures in *Trpv1*^+/+^ mice at 70–140, 165–185, 215–230, and 245–255 min (P < 0.05 vs saline-vehicle) with a peak (1.45 ± 0.17 °C) at 80 min. The icv administration of the TRPV1 antagonist capsazepine significantly promoted LPS-induced hyperthermia at 45–75 and 85–330 min (P < 0.05 vs LPS-vehicle) with a peak (2.35 ± 0.13 °C) at 160 min; however, the administration of capsazepine alone did not have any significant effects on body temperature (P > 0.05 vs saline-vehicle). The icv administration of RTX 500 ng/kg decreased body temperature at 60–235 min (P < 0.05 vs saline-vehicle) with the nadir (−2.63 ± 0.56 °C) at 90 min. However, the RTX-induced decrease in body temperature was significantly larger at 145–330 min in LPS-treated mice (nadir; −3.89 ± 0.91 °C at 115 min, P < 0.05) than in vehicle-treated animals (saline-RTX). Taken together, these results indicate that TRPV1 exerts endogenous antipyretic effects during LPS-induced inflammation.

## Discussion

The mechanisms responsible for the thermoregulatory and inflammatory roles of TRPV1 have been a study subject for the last several decades; however, information regarding these roles in the brain is limited. A large number of studies have demonstrated the peripheral contribution of TRPV1 to thermoregulatory and inflammatory responses. In peripheral tissues, TRPV1 may be continuously activated and act as a suppressor of hyperthermia in order to maintain homeothermy, and, thus, its inactivation by TRPV1 antagonists results in hyperthermia^5^,^10^,^23^. However, the icv administration of TRPV1 antagonists had no effect on body temperature under normal conditions^10^,^13^,^22^. Therefore, hyperthermia induced by TRPV1 antagonists may be due to the inhibition of the TRPV1 signaling cascade in peripheral tissues^5^. In the present study, the icv administration of the TRPV1 agonist RTX induced transient hypothermia under normal conditions, which is consistent with previous findings on capsaicin^8^,^9^, RTX^18^, and arvanil^19^. In the present study, we found that the icv administration of the TRPV1 antagonist capsazepine enhanced the period and magnitude of LPS-induced hyperthermia. Moreover, RTX-induced hypothermia was augmented when combined with the peripheral administration of LPS. Taken together, TRPV1 in the brain appears to be activated under inflammaotry conditons only and acts as an endogenous antipyretic factor to suppress aberrant hyperthemia in homeotherms.

Proinflammatory cytokines such as tumor necrosis factor-α (TNF-α), interleukin-1β (IL-1β) and IL-6 promote the sensitization of TRPV1 in dorsal root ganglion neurons and are involved in the development and maintenance of neuropathic pain^39^. Proinflammatory cytokine levels in serum and the brain were found to be elevated after the peripheral administration of LPS^40^. TNF-α rapidly sensitizes TRPV1 activity and enhances Ca^2+^ influx induced by capsaicin in pulmonary sensory neurons^41^. The levels of the endogenous TRPV1 agonist anandamine in the brain and peripheral tissues were found to be elevated after the peripheral administration of LPS^42^,^43^. Thus, elevations in various cytokines and endogenous agonists may be factors activating TRPV1 in the brain under inflammatory conditions.

We previously revealed that the activation of proinflammatory STAT3 was absent in *Trpv1*^−/−^ mouse brains after the peripheral and icv administration of LPS. The present study showed that the icv administration of the TRPV1 agonist RTX induced the activation of STAT3 under normal conditions. TRPV1 functions in the activation and acquisition of the inflammatory properties of T cells by controlling the T cell receptor signaling complex^44^. The activation of TRPV1 also stimulates extracellular-signal-regulated, c-Jun N-terminal, and p38 mitogen-activated protein kinases and releases the cytokines IL-6 and -8 in a manner that is dependent on extracellular Ca^2+^ levels in dental pulp^45^ and corneal epithelial cells^46^. STAT3 is known to be activated by IL-6 and several growth factors^36^. In contrast, the present study revealed that the icv administration of RTX did not activate proinflammatory NF-κB signaling; however, it was activated in the brain after the administration of LPS^30^,^34^,^36^. A previous study reported that the sensitization of TRPV1 was mediated by TLR4 signaling during treatments and chronic persistent pain in paclitaxel chemotherapy^47^. Recent studies have demonstrated that TRPV1 and TLR4 are expressed by GFAP^+^ NSCs^25^,^32^. However, capsaicin has been shown to inhibit NF-κB activation in macrophages, myelomonoblastic leukemic cells, and dental pulp cells^45^,^48^. Thus, the activation of TRPV1 signaling cascades leads to the activation of proinflammatory STAT3 signaling in the brain under inflammatory conditions.

In the present study, the icv administration of the TRPV1 antagonist capsazepine enhanced the period and magnitude of LPS-induced hyperthermic responses. STAT3 is known to be involved in the transcription of several cytokines in order to maintain brain inflammation^36^. In addition to traditional transcription control, STAT3 regulates cell metabolism by suppressing complexes I and II in mitochondria^49^. The inhibition of mitochondrial STAT3 activation results in reductions in mitochondrial oxidative phosphorylation and cell exocytosis^50^. The icv administration of the STAT3 inhibitor AG490 has been shown to exaggerate LPS-induced hyperthermia, but abrogate LPS-induced reductions in locomotor activity^51^, indicating a dissociation between fever and other components of sickness behavior. Thus, the TRPV1-induced activation of STAT3 signaling may be concerned with inflammatory and thermoregulatory responses during inflammation.

The icv administration of the TRPV1 agonist RTX prominently activated STAT3 in sensory CVOs and moderately activated thermoregulatory hypothalamic regions. The peripheral administration of LPS induced STAT3 activation in GFAP^+^ NSCs in sensory CVOs and GFAP^+^ astrocytes in other brain regions^32^,^34^. Brain cells are equipped with mechanisms that directly detect blood-derived information in sensory CVOs^24^,^52^. The stronger expression of Tlr4 mRNA has been detected in sensory CVOs^30^,^31^ than in other brain regions, and TLR4 is specifically expressed at GFAP^+^ NSCs^32^. In our previous study, TRPV1 was found to be specifically expressed at GFAP^+^ NSCs and the icv administration of RTX induced Fos in GFAP^+^ NSCs in sensory CVOs^25^,^32^. The maintenance of a constant body temperature by homeotherms largely depends on body fluid homeostasis^53^, and TRPV1 is activated by hyperosmolarity^2^. Thus, the results of the present study demonstrated that the TRPV1-dependent STAT3 signaling pathways of the brain were similar to those activated by the peripheral and icv administration of LPS, indicating the importance of TRPV1 signaling in LPS-induced thermoregulatory and inflammatory pathways in the brain.

Previous studies showed that the treatment of neonatal rodents with capsaicin induced brain changes including thinner cortices and increased neuronal density, reminiscent of those reported in schizophrenia^54^,^55^. Brain inflammation induced in neonatal rodents by an injection of LPS or the peripheral administration of *E. coli* elicits schizophrenia-like behavior and structural brain damage in adulthood^56^. Thus, the results of the present study indicate that the ingestion of an excessive amount of capsaicin from food causes brain inflammation and subsequent damage in humans, particularly the young.

## Methods

### Animals

Adult male C57BL/6J strain WT and TRPV1 KO mice (70–105 days old) were housed in a colony room with a 12-h light/dark cycle and given *ad libitum* access to commercial chow and tap water. TRPV1 KO mice were obtained from Dr. D. Julius, University of California, San Francisco, CA^3^. All experiments were performed in accordance with the Guidelines laid down by the NIH and Proper Conduct of Animal Experiments Science Council of Japan. The experimental protocol was approved by the Animal Ethics Experimental Committee of the Kyoto Institute of Technology.

### Administration of LPS and the TRPV1 agonist and antagonist

Stock solutions of RTX (0.7 mg/ml; Carbosynth, Berkshire, UK) and capsazepine (10 mg/ml; Wako pure chemical, Osaka, Japan) were dissolved in 100% ethanol and stored at −80 °C. The stock solutions of RTX and capsazepine were diluted with vehicle solution consisting of pyrogen-free physiological saline (Otsuka Pharmaceutical Co. LTD., Tokushima, Japan) containing 10% Tween 80 and 10% ethanol prior to use. The stock solution of LPS (1 mg/ml; Sigma-Aldrich, 055: type B5) was dissolved in pyrogen-free physiological saline (Otsuka Pharmaceutical Co. LTD.), stored at −80 °C, and diluted with physiological saline prior to use.

In the icv administration protocol, a stainless steel cannula (25-gauge) was implanted in each mouse under anesthesia with chloral hydrate so that its tip laid in the lateral cerebral ventricle (0.3 mm anteroposterior and 1.0 mm lateral to the bregma and 2.5 mm dorsoventral below the skull) using a standard stereotaxic technique (Paxinos and Franklin, 2007). Freely moving mice received the icv administration of RTX (1.25, 2.5, or 5 μg/ml), capsazepine (1 mg/ml), LPS (300 ng/ml), the vehicle solution, and physiological saline using a Model EP-1000E infusion pump (3 μl, 0.5 μl/min, Melquest, Toyama, Japan). Regarding the peripheral LPS stimulation, mice received a single intraperitoneal administration of LPS (5 μg/ml, 0.3 ml).

### Immunohistochemistry

After deep anesthesia with urethane, mice were perfused with PBS (pH 7.4) containing 5 U/ml heparin followed by 4% PFA in 0.1 M PB (pH 7.4). Fixed brains were cryoprotected by 30% sucrose in PBS (pH 7.4) and frozen quickly in Tissue-Tek OCT compound (Sakura Finetechnical, Tokyo, Japan). Sections were obtained by a coronal cut on a cryostat (Leica, Wetzlar, Germany) at a thickness of 30 μm. In single and double immunofluorescent staining, a standard technique was performed on free-floating sections as described in our previous study. In brief, sections were washed with PBS and treated with 25 mM glycine in PBS for 20 min to quench the remaining fixative aldehyde. Sections were preincubated with 5% normal goat serum in PBS containing 0.3% Triton X-100 (PBST) at 4 °C for 24 h and then incubated with the primary antibody in PBST containing 1% ngS at 4 °C for 72 h. The following primary antibodies were used: a mouse monoclonal antibody against HuC/D (Molecular Probes, dilution 1:400); guinea pig antibody against GFAP (dilution 1:500)^32^; rabbit polyclonal antibody against NF-kB p65 (C-20, Santa Cruz Biotechnology, dilution 1,000) and STAT3 (Cell Signaling, dilution 1:2,000). After several washes with PBST, they were further incubated with an Alexa 488- or 594-conjugated secondary goat antibody (Jackson ImmunoResearch, dilution 1:400).

### Confocal observation and quantification

In the confocal microscopic observations, coverslips were sealed with Vectashield (Vector Labs, Burlingame, CA) and observations were performed using a laser-scanning confocal microscope (LSM-510, Carl Zeiss). We selected at least 5 sections per animal from the OVLT (between bregma 0.50 and 0.62 mm) and 7 sections per animal from the MnPO (between the bregma 0.14 and 0.38 mm), POA (between the bregma 0.14 and −0.10 mm), SFO (between the bregma −0.58 and −0.82 mm), and AP and Sol (between the bregma −7.48 and −7.64 mm) according to the mouse brain atlas^57^. In order to perform a quantitative analysis, confocal images were obtained under the same pinhole size, brightness, and contrast setting. We saved images (1,024× 1,024 pixels) as TIF files by employing the Zeiss LSM image browser for Windows and arranged them using Photoshop CC. In quantitative analyses, the total area of the OVLT, SFO, and AP was measured using WinRoof, an image analyzing system (Mitani Corporation, Fukui, Japan). The numbers of STAT3- and NF-kB-positive nuclei in GFAP-labeled astrocytes and HuC/D-labeled neurons were counted using WinRoof, the threshold intensity of which was set to include measurement profiles by visual inspections and was kept constant. An analysis of all images was performed such that the experimenter was blind to the treatment group. Differences from the vehicle were assessed using a significance level of P < 0.05 (Student’s *t*-test).

### Measurement of body temperature

Mice were anesthetized with chloral hydrate, implanted intraperitoneally with a transponder (G2 E-mitter) that recorded core body temperature, and were then housed at an ambient temperature of 25 °C under a 12-h light/dark cycle (lights on at 7:00 A.M.). Mice were kept for at least 1 week after the implantation of the telemeter. The administration of RTX and LPS was performed between 11.00 h and 12.00 h. Abdominal temperature and gross locomotor activity were measured by biotelemetry at 5-min intervals and plotted at 10-min intervals over a period of 60 min before and 360 min after the treatment. Data were acquired and fed to a computer using Vital View software (VitalView series 4000).

## Additional Information

**How to cite this article**: Yoshida, A. *et al*. TRPV1 is crucial for proinflammatory STAT3 signaling and thermoregulation-associated pathways in the brain during inflammation. *Sci. Rep*. **6**, 26088; doi: 10.1038/srep26088 (2016).

## Supplementary Material

Supplementary Information

## Figures and Tables

**Table 1:** STAT3 activation in GFAP^+^ NSCs and astrocytes in the mouse brain by peripheral and icv administration of LPS and icv administration of RTX.

Brain regions	Trpv1^+/+^		Trpv1^−/−^		Trpv1^+/+^		Trpv1^−/−^		Trpv1^+/+^		Trpv1^−/−^	
	Vehicle ip	LPS ip	Vehicle ip	LPS ip	Vehicle icv	LPS icv	Vehicle icv	LPS icv	Vehicle icv	RTX icv	Vehicle icv	RTX icv
Forebrain												
Cerebrum	−	−	−	−	−	−	*	*	−	−	−	−
Cerebellum	−	−	−	−	−	−	*	*	−	−	−	−
Olfactory bulb	−	−	−	−	−	−	*	*	−	−	−	−
Limbic system												
Hippocampus	−	−	−	−−	−	−	*	*	−	−	−	−
Fimbria	−	−/+	−	−	−	−/+	*	*	−	−/+	−	−
Ventral hippocampal commissure	−	++	−	−/+	−	++	*	*	−	++	−	−
Hypothalamus												
Arcuate hypothalamic nucleus	−	+	−	−	−	+	*	*	−	+	−	−
Lateral septal nucleus	−	−	−	−	−	−	*	*	−	−	−	−
Median preoptic nucleus	−	+	−	+	−	+++	−	−	−	+++	−	−
Preoptic area	−	+	−	−	−	+++	−	−	−	++	−	−
Periventricular hypothalamic nucleus	−	+	−	−	−/+	+	*	*	−/+	++	−/+	−/+
Paraventricular hypothalamic nucleus	−	+	−	−	−	+	*	*	−	++	−	−
Suprachiasmatic nucleus	−	−	−	−	−	−	*	*	−	−/+	−	−
Supraoptic nucleus	−	+	−	+	−	++	*	*	−	−/+	−	−
Supramammillary nucleus	−	−	−	−	−	−	*	*	−	−	−	−
Ventromedial hypothalamic nucleus	−	−	−	−	−	−	*	*	−	−	−	−
Circumventriculae organs												
Vascular organ of the lamina terminalis	−	++	−	+	−	+++	−	−	−	+++	−	−
Subfornical organ	−	+++	−	+	−	+++	−	−	−	+++	−	−
Median eminence	−/+	+	−	+	−	+	−	−	−	+	−	−
Area postrema	−	+++	−	+	−	+++	−	−	−	+++	−	−
Brainstem												
Solitary nucleus	−	++	−	++	−	++	−	−	−	+++	−	−
10N	−	−	−	−	−	−	*	*	−	−	−	−
12N	−	−	−	−	−	−	*	*	−	−	−	−
Other nonneuronal cells												
Meninges	−	++	−	++	−/+	++	−/+	−/+	−/+	+	−/+	−/+

Relative values of nuclear STAT3 immunoreactivity are given as estimates of the density of nuclear STAT3 labeling. Nuclear STAT3 immunoreactivity was examined 2 hr after administration of LPS, RTX, or an equivalent volume of vehicle. A five point scale was used to rate nuclear translocation of STAT3: − none; −/+ none/week; + week; ++ moderate; +++ strong; *Not examined.
