# Supplementary material for: TRPV1 is crucial for proinflammatory STAT3 signaling and thermoregulation-associated pathways in the brain during inflammation
Source: Sci Rep. 2016 May 18;6:26088. doi: 10.1038/srep26088 (PMC4870621; doi:10.1038/srep26088)
Supplement: Supplementary Information [file srep26088-s1.pdf]

Supplementary information

TRPV1 is crucial for proinflammatory STAT3 signaling and thermoregulation-associated pathways in the brain during inflammation

Ayaka Yoshida[1], Eriko Furube[1], Tetsuya Mannari[1], Yasunori Takayama[2], Hiroki Kittaka[2], Makoto Tominaga[2], AND Seiji Miyata[1, *]

[1]*Department of Applied Biology, Kyoto Institute of Technology, Matsugasaki, Sakyo-ku, Kyoto 606-8585, Japan.*

[2]*Division of Cell Signaling, Okazaki Institute for Integrative Bioscience, (National Institute for Physiological Sciences), National Institute of Natural Sciences, Okazaki, Aichi 444-8787, Japan.*

Fig. S1

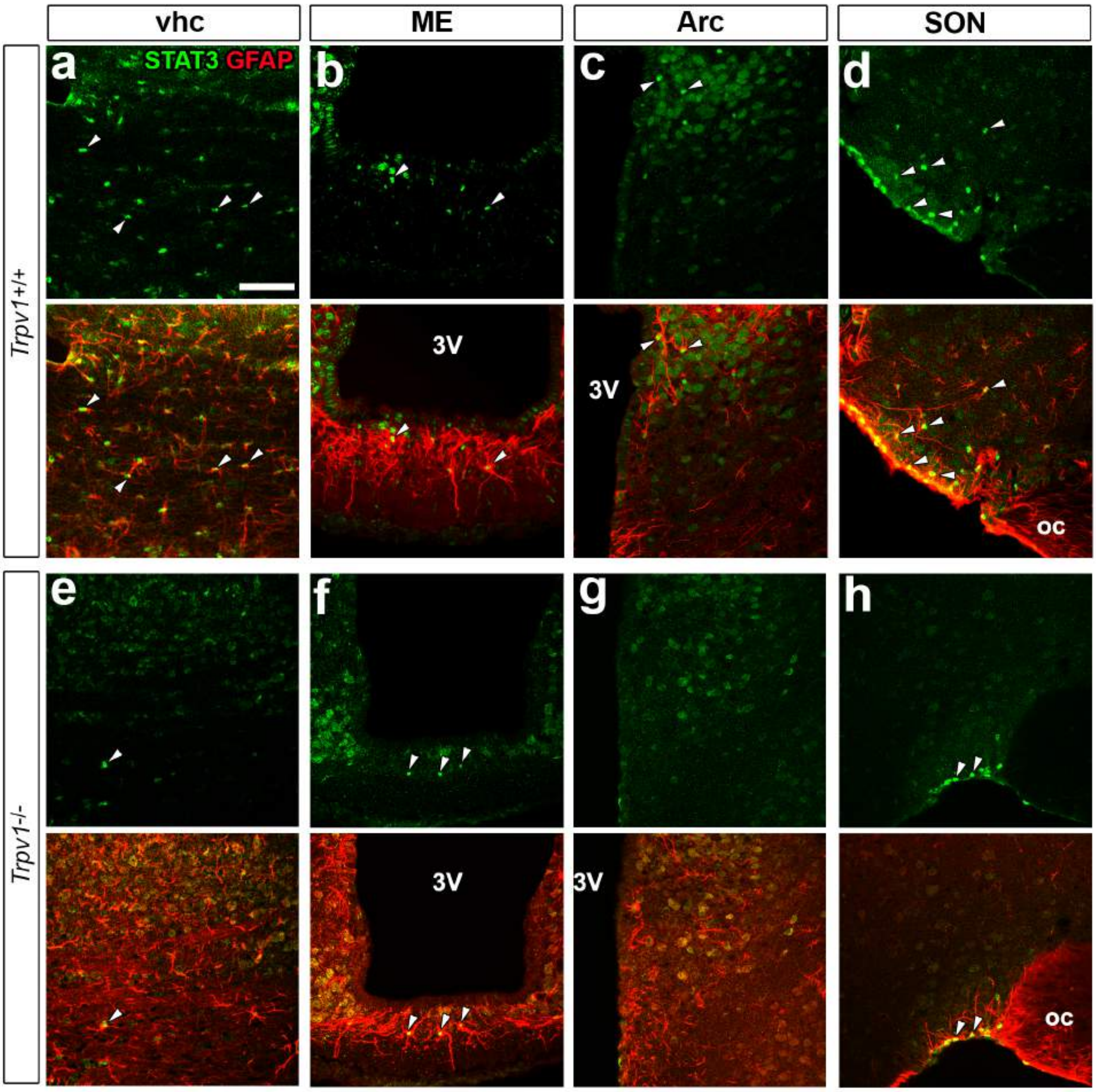

Fig. S1

A deficiency in STAT3 signaling activation in GFAP$^+$ astrocytes in the vhc, ME, Arc, and SON in *Trpv1*$^{-/-}$ mice following the peripheral LPS stimulation. C57BL/6J mice received an intraperitoneal administration of 50 µg/kg LPS and were sacrificed for STAT3 immunohistochemistry. The nuclear translocation of STAT3 (arrowheads) was detected in GFAP$^+$ astrocytes in the vhc, ME, Arc, and SON in *Trpv1*$^{+/+}$ mice (a-d) 2 hr after the peripheral LPS stimulation, while it was rarely observed in *Trpv1*$^{-/-}$ mice (e-h). oc, optic chiasma; 3rd ventricle, 3V. Scale bar = 50 µm.

Fig. S2

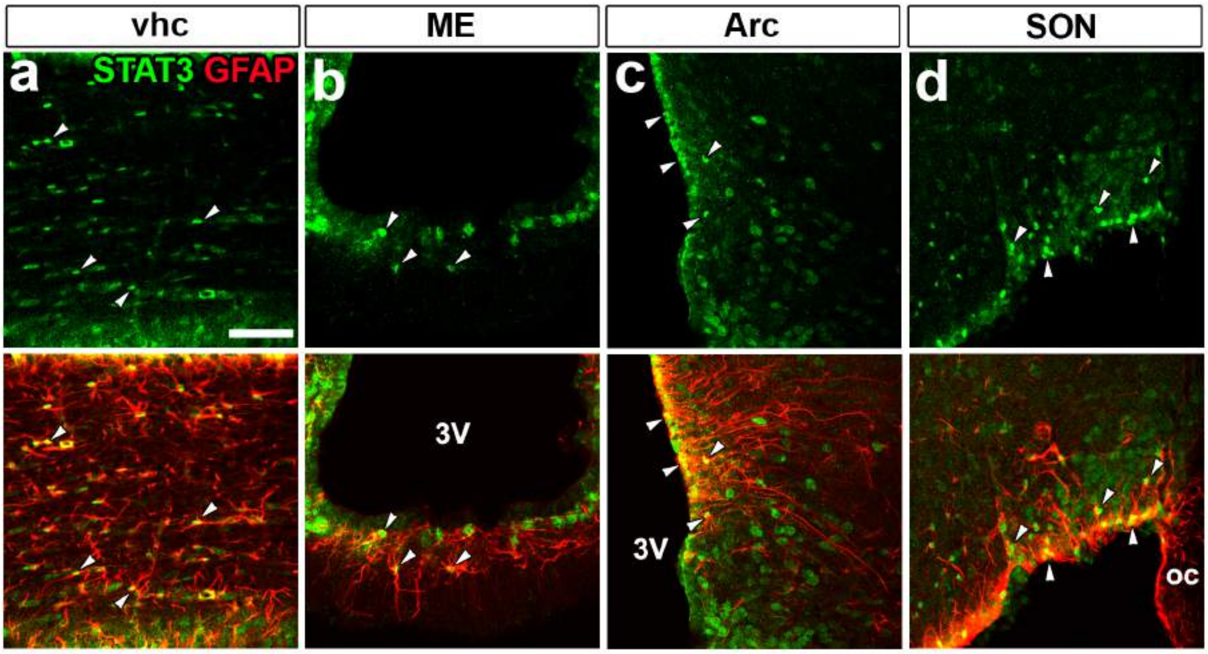

Fig. S2

The nuclear translocation of STAT3 occurred in GFAP⁺ astrocytes in the vhc, ME, Arc, and SON in *Trpv1*$^{+/+}$ mice after the brain infusion of LPS. C57BL/6J mice received an icv administration of 30 ng/kg LPS and were sacrificed for STAT3 immunohistochemistry. The nuclear translocation of STAT3 (arrowheads) was detected in GFAP⁺ astrocytes in the vhc, ME, Arc, and SON in *Trpv1*$^{+/+}$ mice 2 hr after the central LPS administration. oc, optic chiasma; 3rd ventricle, 3V. Scale bar = 50 μm.

-/-

Fig. S3

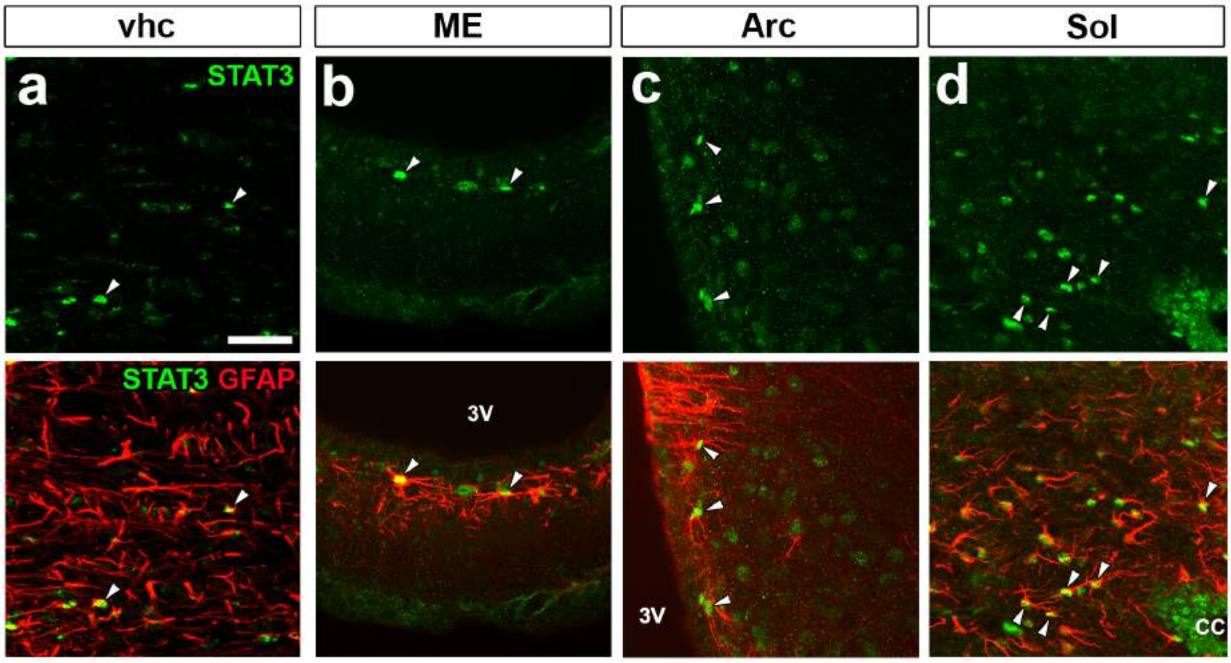

Fig. S3

The activation of TRPV1 induced the nuclear translocation of STAT3 in GFAP$^+$ astrocytes in the vhc, ME, Arc, and SON in *Trpv1*$^{+/+}$ mouse brains. C57BL/6J mice received an icv administration of 500 ng/kg RTX and were sacrificed for STAT3 immunohistochemistry. The nuclear translocation of STAT3 (arrowheads) was detected in GFAP$^+$ astrocytes in the vhc, ME, Arc, and SON in *Trpv1*$^{+/+}$ mice 2 hr after the central LPS administration. oc, optic chiasma; 3rd ventricle, 3V. Scale bar = 50 μm.

Fig. S4

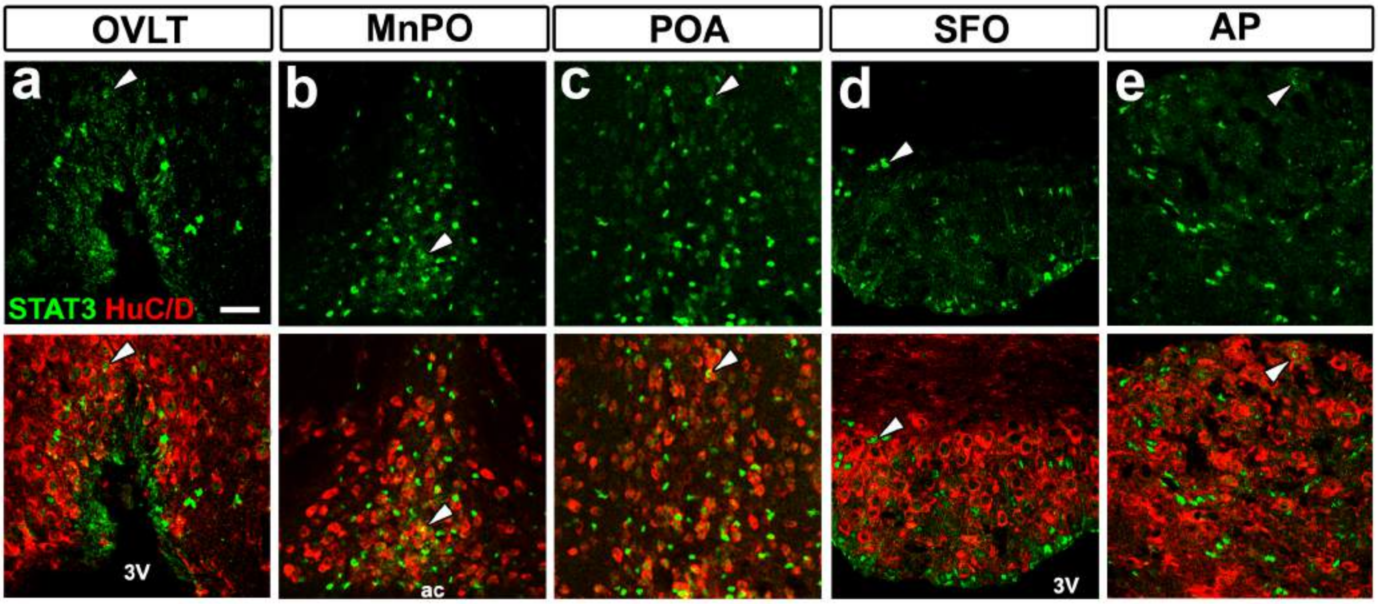

Fig. S4

The activation of TRPV1 induced the nuclear translocation of STAT3 (arrowheads) in a small number of HuC/D$^+$ neurons in sensory CVOs and thermoregulatory hypothalamic subregions in *Trpv1*$^{+/+}$ mice. C57BL/6J mice received an icv administration of 500 ng/kg RTX and were sacrificed for STAT3 immunohistochemistry. Although the nuclear translocation of STAT3 occurred in sensory CVOs and thermoregulatory hypothalamic subregions, STAT3$^+$ nuclei were rarely observed in HuC/D$^+$ mature neurons. ac, anterior commissure; 3rd ventricle, 3V. Scale bar = 50 μm.

Fig. S5

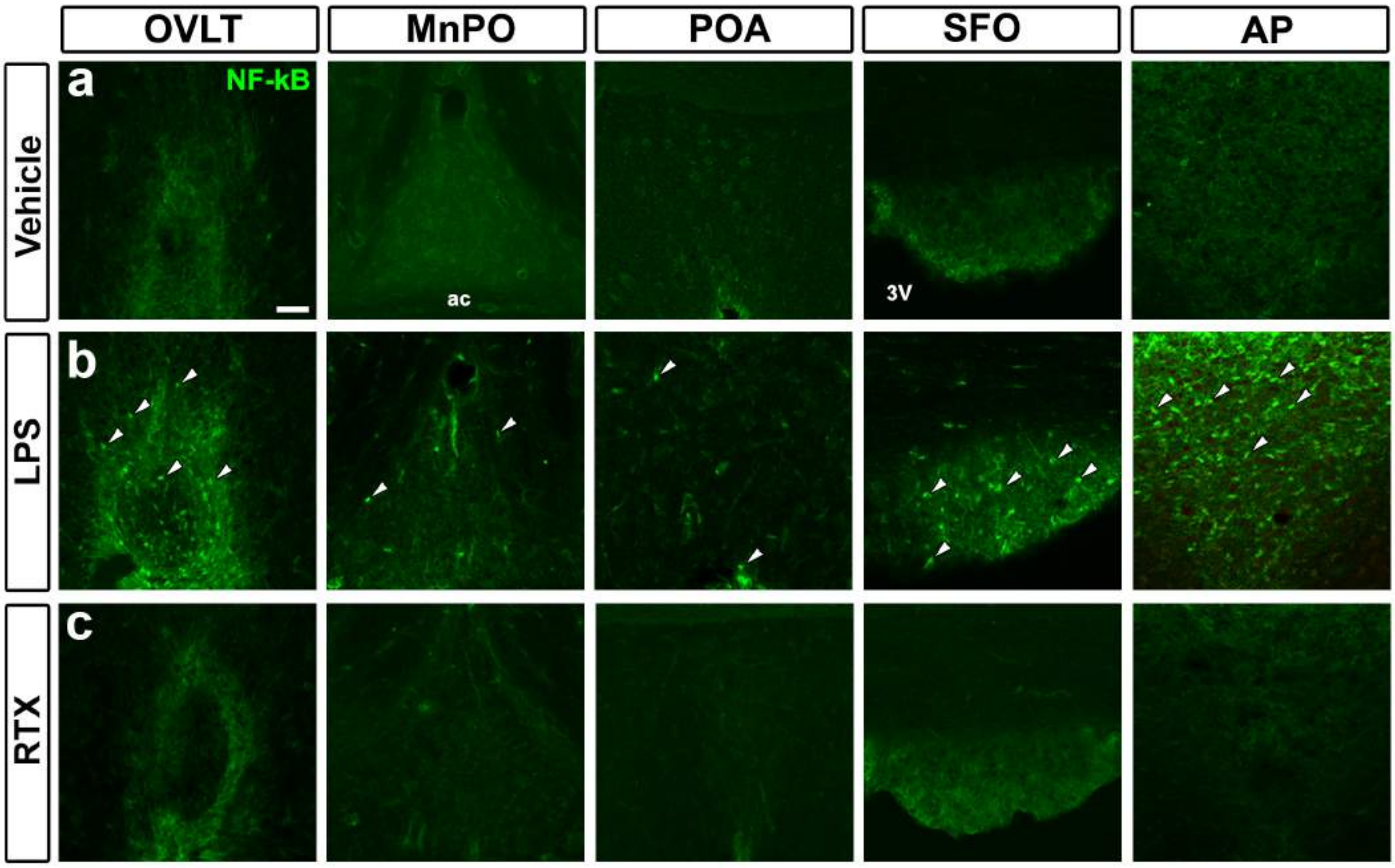

Fig. S5

The activation of TRPV1 induced almost no nuclear translocation of NF-kB (arrowheads) in sensory CVOs or thermoregulatory hypothalamic subregions in *Trpv1*$^{+/+}$ mice. C57BL/6J mice received an icv administration of 500 ng/kg RTX and were sacrificed for NF-kB immunohistochemistry. Although the nuclear translocation of NF-kB was observed in sensory CVOs and thermoregulatory hypothalamic subregions upon the peripheral LPS stimulation, nuclear translocation was never detected after the central administration of RTX. ac, anterior commissure; 3rd ventricle, 3V. Scale bar = 50 μm.

Supplementary Table

Treatment	Changes in temperature		Statistical significance (P < 0.05)	n
	Peak	Nadir		
**a**				
Trpv1$^{+/+}$ vehicle	1.50 ± 0.60 (20 min)	0.26 ± 0.67 (90 min)		5
Trpv1$^{+/+}$ RTX 125 ng/kg	1.44 ± 0.24 (15 min)	0.05 ± 0.30 (105 min)	None (vsTRPV1+/+ vehicle)	5
Trpv1$^{+/+}$ RTX 250 ng/kg	1.40 ± 0.23 (10 min)	-1.38 ± 0.94 (50 min)	35 ~ 45 min (vs TRPV1+/+ vehicle)	5
Trpv1$^{+/+}$ RTX 500 ng/kg	0.43 ± 0.52 (10 min)	-2.79 ± 0.28 (45 min)	25~140 min (vsTRPV1+/+ vehicle)	5
			20~95 min (vsTRPV1-/-, RTX 500 ng/kg)	
Trpv1$^{-/-}$ RTX 500 ng/kg	1.50 ± 0.60 (20 min)	-0.66 ± 0.32 (135 min)		5
**b**				
Saline-Vehicle	0.79 ± 0.30 (50 min)	0.11± 0.18 (325 min)		7
LPS-Vehicle	1.45 ± 0.17 (80 min)	0.47 ± 0.31 (30 min)	70~140, 165~185, 215~230, 245~255 min (vs Saline-Vehicle)	8
Saline-RTX	1.02 ± 0.26 (35 min)	-2.63 ± 0.56 (90 min)	60~235 min (vs Saline-Vehicle)	5
LPS-RTX	0.48± 0.65 (20 min)	-3.89 ± 0.91 (115 min)	45~330 min (vs Saline-Vehicle)	5
			50~330 min (vs LPS-Vehicle)	
			145~330 min (vs Saline-RTX)	
Saline-Capsazepine	0.89 ± 0.27 (45 min)	0.46 ± 0.33 (285 min)	None (vs Saline-Vehicle)	5
LPS-Capsazepine	2.35 ± 0 .13 (160 min)	1.50 ± 0.60 (20 min)	45~330 min (vs Saline-Vehicle)	7
			45~75, 85~330 min (vs LPS-Vehicle)	
			55~330 min (vs Saline-CPZ)	
